# Trends in Gastroschisis in the State of Paraná, Brazil: A Study of Incidence, Mortality, and Associated Factors (2013–2024)

**DOI:** 10.3390/ijerph23030387

**Published:** 2026-03-18

**Authors:** Paulo Acácio Egger, Matheus Henrique Arruda Beltrame, Makcileni Paranho de Souza, Cristiane de Oliveira Riedo, Amanda de Carvalho Dutra, Wagner Sebastião Salvarani, Sandra Marisa Pelloso, Maria Dalva de Barros Carvalho

**Affiliations:** 1 Department of Medicine, State University of Maringá, Maringá 87083-240, Brazil; matheushbeltrame@gmail.com; 2 Health Sciences Program, State University of Maringá, Maringá 87030-230, Brazil; mpsouza@uem.br (M.P.d.S.); coriedo@uem.br (C.d.O.R.); amandacarvalhodutra@gmail.com (A.d.C.D.); enf.wagnersalvarani@gmail.com (W.S.S.); smpelloso@uem.br (S.M.P.); mdbcarvalho@uem.br (M.D.d.B.C.)

**Keywords:** gastroschisis, fatality, lethality, prevalence, incidence, abdominal wall, mortality

## Abstract

**Highlights:**

**Public health relevance—How does this work relate to a public health issue?**

- This study with population data is fundamental for public health because it acts as an indicator of the quality of maternal and child care and the effectiveness of surveillance policies in Paraná.
- The work qualifies the use of national systems, which are: the Live Birth Information System (SINASC) and the Mortality Information System (SIM), which are important national database systems.

**Public health significance—Why is this work of significance to public health?**

- This work is important for public health because it allows for an accurate diagnosis of conditions related to children born with gastroschisis and its lethality.
- The 36.5% lethality rate associated with gastroschisis is a critical warning sign.

**Public health implications—What are the key implications or messages for practitioners, policy makers and/or researchers in public health?**

- The healthcare system needs an adequate network that enables prenatal care and delivery of children with gastroschisis in hospitals that are referral centers for neonatal intensive care and efficient pediatric surgery services.
- With improved care for pregnant women and children with gastroschisis, mortality rates can decrease.

**Abstract:**

This population-based study aimed to analyze the annual incidence and case fatality trends, and the clinical-epidemiological profile of gastroschisis in the state of Paraná, Brazil, between 2013 and 2024. Specifically, temporal trends in annual incidence and mortality rates related to gastroschisis were examined. Maternal, gestational, and neonatal characteristics were analyzed. Data from the Live Birth Information System and the Mortality Information System were analyzed using polynomial regression modeling. During the study period, 1,798,727 live births were recorded, including 491 cases of gastroschisis and 179 related deaths. The mean incidence was 2.73 per 10,000 live births. A significant 39.5% decrease over the study period was observed (*p* < 0.001). The case fatality rate was 36.5%. The mothers of children with gastroschisis were: young mothers (<25 years old; 77%), with low education (87.7%) and no partner (59.1%). High frequencies of cesarean deliveries (84.3%), prematurity (57.3%), low birth weight (63.7%), and low Apgar scores were also observed. The profiles of the mothers and children at birth were unfavorable when compared to the population of live births. Gastroschisis incidence in Paraná declined significantly from 2013 to 2024. While the annual incidence showed a decreasing trend, mortality fluctuated. The persistently high case fatality rate underscores the need for public policies focused on prenatal care and specialized neonatal management.

## 1. Introduction

Gastroschisis is a congenital defect of the paraumbilical abdominal wall closure, most often occurring on the right side, without a covering membrane but with an intact umbilical cord. The defect is characterized by the extrusion of the abdominal viscera from beyond the peritoneal cavity [1].

The prevalence of congenital anomalies typically varies over time and across different regions and countries [2,3,4]. Since the 1960s, gastroschisis has shown a significant global increase. Historically, a “gastroschisis pandemic” was noted in the literature due to increases of up to 300% over two decades [5]. In contrast, recent data from various health systems suggest trend reversal, with the prevalence of gastroschisis declining over the past 15 years [2,4,6]. These fluctuations in prevalence—both increases and decreases—are difficult to explain, given that gastroschisis has a complex multifactorial etiology as described by several studies [4,7,8]. Several etiological and pathology-associated factors have been proposed, such as: young pregnant women [9], genetic predisposition [7], medication use [10], drug use (tobacco, alcohol, marijuana, cocaine and crack) [11], maternal infections [12], electromagnetic fields [8] and pesticides [13].

Patients with gastroschisis experience high morbidity, often requiring multiple operations and prolonged hospital stays [14]. The costs associated with treating affected children are substantial and represent a significant burden to healthcare systems. The substantial costs associated with the treatment of children with gastroschisis underscore the importance of studying its incidence, associated characteristics, and mortality [15,16,17].

Over the past sixty years, gastroschisis-related mortality has declined in developed and developing countries. This reduction, however, has not been observed in low-income countries. Developed countries currently report lethality rates close to 5% [18]. Developing nations show intermediate outcomes, while the mortality rate in low-income countries ranges from 75% to 100% [3,19,20].

Despite the relevance of this topic, there is a notable gap in comprehensive longitudinal data from southern Brazil. This study population-based analysis to simultaneously assess incidence, lethality, and maternal-infant factors associated with gastroschisis in the state of Paraná. Paraná is located in southern Brazil, with an estimated population of 11,890,517 in 2025, and ranks fourth in the country according to the Human Development Index (HDI) [21].

For the epidemiological assessment of gastroschisis, temporal trend analysis is of great value in identifying variations in annual incidence. Investigating these variations allows not only the identification of changes in the risk profile of populations, but also the evaluation of the effectiveness of perinatal care policies and the support for planning specialized health resources. This study aimed to analyze the temporal trends in the annual incidence and case fatality rate of gastroschisis in Paraná between 2013 and 2024, and to characterize the clinical-epidemiological profile of affected mothers and newborns.

## 2. Materials and Methods

### 2.1. Study Design, Setting, and Period

This is a population-based observational study with a temporal trend design, analyzing data on live births diagnosed with gastroschisis in the state of Paraná, Brazil, and its health macroregions (MRs), between 2013 and 2024.

Data on live births, hospitalizations, and mortality associated with gastroschisis were obtained from the official website of the Department of Informatics of the Brazilian Unified Health System (DATASUS) [22]. The databases accessed were the Live Birth Information System (SINASC) and the Mortality Information System (SIM). Considering the territorial size of Brazil and the presence of a unified health system, Brazilian population databases can be considered among the largest in the world [23]. Data collection was conducted in 2025 in the city of Maringá, Paraná [22,24].

### 2.2. Population and Inclusion/Exclusion Criteria

This study analyzed hospitalization data for newborns diagnosed with gastroschisis (ICD-10 code Q79.3), classified according to the International Classification of Diseases, 10th Revision (ICD-10).

Variables relating to maternal, pregnancy, and neonatal characteristics were analyzed. Specifically, the following variables were maternal age (10–19 years, 20–24 years, and over 25 years), maternal marital status, maternal education level, number of prenatal visits, type of pregnancy (singleton or multiple), gestational age (<37 weeks or ≥37 weeks), type of delivery (cesarean or vaginal), infant race/ethnicity, Apgar score at one minute, Apgar score at five minutes, birth weight (<2500 g or ≥2500 g), sex, annual incidence rate, and mortality rate.

### 2.3. Study Protocol

For the rate calculations, hospitalizations were selected based on data from the SINASC database for Paraná, in which gastroschisis was recorded as a diagnosis [14]. Mortality was determined based on data from the SIM database, where Q79.3 (gastroschisis) was listed as the underlying cause of death [24]. Variables were grouped into three categories: maternal (age, marital status, and educational level), gestational (number of prenatal visits, type of pregnancy, and mode of delivery), and neonatal (sex, race/ethnicity, birth weight, gestational age, and Apgar scores at 1 and 5 min). Relative frequencies (percentages) of the variables, the annual incidence, and the mortality rate were calculated. Both the annual incidence and mortality rate were calculated as the ratio between the number of events and the total number of live births among residents, multiplied by a constant of 10,000.

### 2.4. Data Analysis and Statistical Methods

Trends in mortality and annual incidence rates were analyzed using polynomial regression modeling, with the rates considered as dependent variables (y) and year as the independent variable (x). To avoid autocorrelation, the centered year variable (x–2018) was used. The time series was smoothed using a three-point moving average. Polynomial regression models tested included linear (y = β_0_ + β_1×1_), quadratic (y = β_0_ + β_1×1_ + β_2×2_), and cubic (y = β_0_ + β_1×1_ + β_2×2_ + β_3×3_). A trend was considered statistically significant when the estimated model had a *p*-value < 0.05. Model selection was further guided by the scatter plot analysis, the coefficient of determination (***R***^2^), and residual analysis. When all criteria were significant for more than one model and ***R***^2^ values were similar, the simpler model was preferred. Trend and polynomial regression analyses were performed for the entire state of Paraná as well as for its health MRs. Paraná is divided into four health MRs: Northern, Northwestern, Eastern, and Western. The southern part of the state is incorporated into the eastern and western regions. To compare the proportions of categorical variables, Pearson’s chi-square test was used. The significance was set at 5%. Analyses were performed using Microsoft Excel (version 2024) and Epi Info (version 7.2.7.0).

### 2.5. Ethical Aspects

As this study was based on secondary data available in the public domain, it was exempt from review by the Permanent Committee for Ethics in Research Involving Human Beings, in accordance with Resolution No. 510, dated 7 April 2016, of the Brazilian National Health Council [15].

## 3. Results

### 3.1. Annual Incidence and Mortality/Case Fatality

Between 2013 and 2024, 1,798,727 live births were recorded in Paraná. Among these, 491 were diagnosed with gastroschisis. During the same period, 179 gastroschisis-related deaths (36.5%) were reported (Table 1).

**Figure 1:**
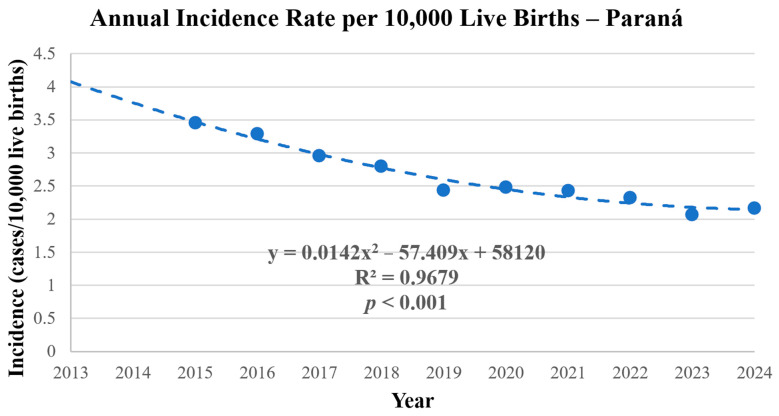
Annual Incidence of Gastroschisis in Paraná, Brazil, 2013–2024. Source: SINASC/SUS, 2013–2024.

The mean annual incidence of gastroschisis in Paraná during the study period was 2.73 cases per 10,000 live births, ranging from 3.92 (in 2013) to 2.04 (in 2021) (Table 1; Figure 1). Polynomial regression analysis confirmed a significant downward trend in annual incidence (*p* < 0.001), with a total reduction of 39.5% over the study period. A marked decline in incidence was observed between 2013 and 2021, followed by a stabilization from 2022 to 2024 (Table 1 and Table 2; Figure 1).

Regarding clinical outcomes, 179 deaths were recorded during the study period, resulting in a mean case fatality rate of 36.5%. The case fatality rate exhibited a fluctuating pattern, with the lowest rate observed in 2020 (16.7%) and the highest in 2023 (48.3%). Among the deaths, 97.7% occurred within the first year of life. The age of death ranged from one day old to four years old (Table 1).

**Figure 2:**
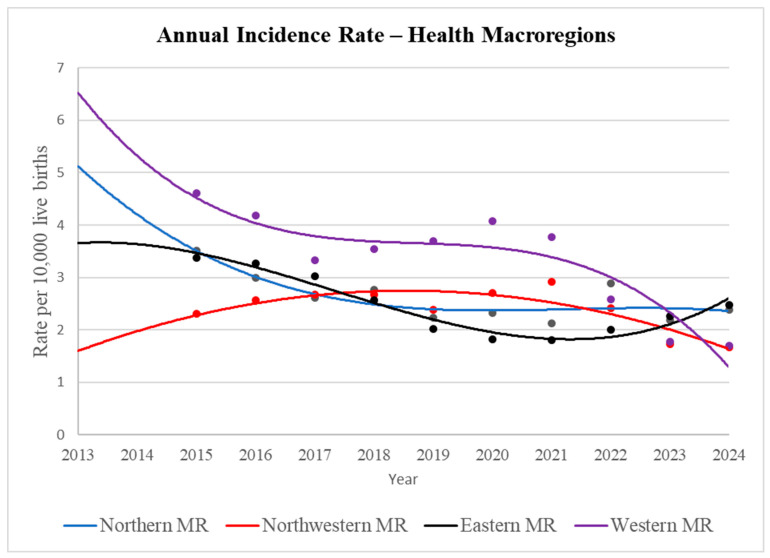
Annual Incidence of Gastroschisis in the Health Macroregions of Paraná, Brazil, 2013–2024. Source: SINASC/SUS, 2013–2024.

Polynomial regression analysis revealed that the health MRs of Paraná exhibited distinct patterns of annual gastroschisis incidence. Most regions (Northern, Eastern, and Western) showed statistically significant trends. Specifically, the Northern and Western MRs demonstrated a significant decline in incidence, while the Eastern MR displayed a more complex bimodal trend—initially decreasing, then increasing (*p* < 0.05 for both). In contrast, the Northwestern MR showed an initial increase followed by a decrease in incidence, though this trend was not statistically significant (Table 2; Figure 2).

**Figure 3:**
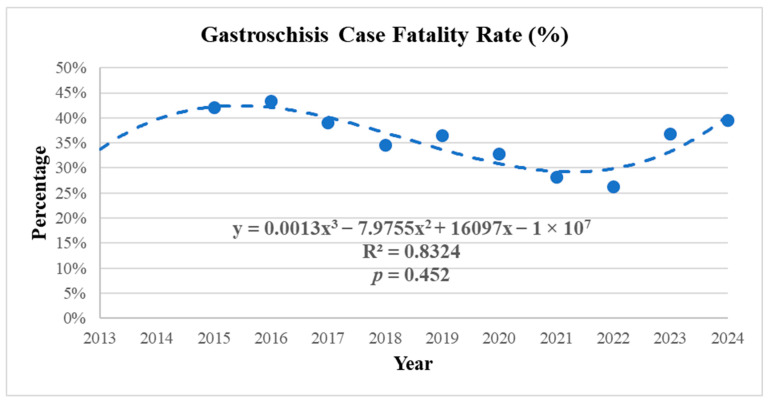
Trend in Gastroschisis Case Fatality Rate in Paraná, Brazil, 2013–2024. Source: SINASC/SUS, 2013–2024.

Polynomial regression analysis applied to the case fatality rate (percentage of deaths relative to the number of cases per year) demonstrated a stable trend with fluctuations and no statistical significance (Table 1 and Table 2; Figure 3).

### 3.2. Maternal Characteristics: Maternal Age, Marital Status, Educational Level, and Prenatal Care Visits

The annual incidence of gastroschisis increased significantly among mothers under 25 years of age (5.61 cases per 10,000 live births). The incidence was even higher among those under 20 years of age (8.09 cases per 10,000) (Table 3). Relative risk (RR) analysis and Pearson’s chi-square test comparing mothers under 25 versus those aged 25 or older revealed a relative risk of 5.59 (*p* < 0.001; 95% confidence interval [95% CI]: 4.53–6.90).

This result indicated that mothers under 25 years of age were 5.6 times more likely to have a child with gastroschisis than those 25 years and over (Table 3).

A significant association was observed between maternal marital status and the occurrence of gastroschisis. Mothers without a partner had a relative risk (RR) of 1.38 (95% CI: 1.29–1.49; *p* < 0.001) compared to those with a partner. This indicated that women without a partner had a 38% higher risk of giving birth to a child with this malformation. Additionally, the absolute risk difference was 16.5%, highlighting greater vulnerability in this group (Table 3).

The analysis also revealed that low educational attainment (less than 12 years of schooling) is associated with a higher risk of gastroschisis. The relative risk was 2.49 (95% CI: 1.90–3.26; *p* < 0.001), indicating that mothers with lower education levels are 2.49 times more likely to have children with this malformation compared to the reference group (Table 3).

Babies of mothers who had fewer than seven prenatal care visits had a significantly greater rate of gastroschisis. The relative risk was 1.61 (95% CI: 1.38–1.87; *p* < 0.001), suggesting that mothers of affected children were 61% more likely to have had inadequate prenatal care compared to mothers of unaffected children. The absolute risk difference was 9.6%, confirming the greater probability of insufficient prenatal visits in the case group (Table 3).

### 3.3. Pregnancy Characteristics: Type of Pregnancy, Gestational Age, and Mode of Delivery

The type of pregnancy did not reveal a significant association with the occurrence of gastroschisis. The relative risk was 0.97 (95% CI: 0.53–1.77; *p* = 0.94), indicating that twin pregnancies do not carry a higher risk for malformation compared to singleton pregnancies (Table 4).

A strong association was observed between gastroschisis and prematurity. While only 10.7% of the general live birth population was born before 37 weeks of gestation, this percentage rose to 57.3% among gastroschisis cases. The calculated relative risk was 5.33 (95% CI: 4.93–5.76; *p* < 0.001), indicating that infants with gastroschisis are five times more likely to be born preterm. The absolute risk difference of 46.5% further highlighted the high probability of early delivery in these cases (Table 4).

Regarding the mode of delivery, statistical analysis demonstrated a significant association between gastroschisis and cesarean section. The relative risk for cesarean delivery was 1.32 (95% CI: 1.27–1.38; *p* < 0.001), indicating that infants with gastroschisis have a 32% higher risk of being born via this surgical procedure compared to the general population. The absolute risk difference was 20.8%, reinforcing the increased likelihood of surgical intervention in such cases (Table 4).

### 3.4. Neonatal Characteristics: Race/Ethnicity, 1-Minute Apgar Score, 5-Minute Apgar Score, Birth Weight, and Sex

Considering race/ethnicity, a predominance of White newborns was observed (74.5%), a proportion similar to that of the general live birth population (73.9%). Statistical analysis confirmed that there were no significant differences among racial/ethnic categories (*p* > 0.05), indicating that race/ethnicity was not a risk factor for gastroschisis in this sample (Table 5).

Neonatal vitality at the first minute of life showed severe compromise among gastroschisis cases. While only 12.1% of newborns in the general population presented an Apgar score below 8, this proportion rose to 49.8% among those with the malformation. Statistical analysis confirmed a relative risk of 4.09 (95% CI: 3.74–4.47; *p* < 0.001), indicating that infants with gastroschisis are four times more likely to exhibit low vitality at one minute of life. The absolute risk difference of 37.6% underscores the need for specialized care and immediate neonatal resuscitation for these patients (Table 5).

Evaluation at five minutes of life revealed an even stronger association with gastroschisis. The calculated relative risk was 9.30 (95% CI: 7.78–11.12; *p* < 0.001), indicating that infants with the malformation have a nine-fold increased risk of maintaining Apgar scores below 8 at the fifth minute than that of the control group. Combined with an absolute risk difference of 17.7%, the high relative risk highlights the persistence of immediate clinical compromise in these newborns (Table 5).

Moreover, gastroschisis was strongly associated with low birth weight (<2500 g). While only 8.7% of the general live birth population presented with low birth weight, this proportion rose sharply to 63.7% among affected cases. Statistical analysis revealed a relative risk of 7.29 (95% CI: 6.82–7.80; *p* < 0.001), indicating that infants with the malformation were seven times more likely to be born with insufficient weight. The absolute risk difference of 55.0% confirmed the high prevalence of low birth weight associated with this condition (Table 5).

Furthermore, the sex distribution of gastroschisis was similar (50.7% male and 49.3% female) to that observed in the general live birth population. Statistical analysis revealed no significant association between sex and the presence of the malformation, with a relative risk of 0.99 (95% CI: 0.90–1.08; *p* = 0.82) (Table 5).

## 4. Discussion

This population-based study in the state of Paraná, Brazil, to investigate the annual incidence and mortality of gastroschisis. Paraná is located in the southern region of Brazil, one of the most developed areas in the country. The state has a Human Development Index (HDI) of 0.769, which is classified as high human development. This study advances the field by integrating temporal trend analysis with maternal, gestational, and neonatal characteristics, in addition to examining mortality outcomes associated with the malformation [21].

The findings revealed a statistically significant 39,5% reduction in the trend of annual gastroschisis incidence in Paraná from 2013 to 2024. Globally, the incidence and prevalence of this malformation have shown divergent patterns since the year 2000 [4,8,25]. While the first decade of the 21st century was marked by an upward trend in several regions worldwide [5,20,25,26], more recent data have demonstrated a general decline. Among the available studies, a pioneering study conducted in Liaoning Province, China, in 2016 was the first to report this decline [27]. This result was later corroborated by other investigations [4,28]. The results from this present study are consistent with these previous findings. Notably, evidence from the United States suggests that the reduction in gastroschisis prevalence may be strongly linked to a decline in pregnancy rates among women under 20 years of age [28]. In Brazil, the Ministry of Health reported, through a technical note, a decrease in adolescent births from 18.9% to 11.9% over the past decade [29]. This reduction in adolescent pregnancies may be positively associated with the decline in gastroschisis cases, although further scientific studies are needed to confirm this hypothesis [28].

Stratified analysis by the four health MRs of Paraná revealed decreasing trends in gastroschisis rates (per 10,000 live births), with statistical significance in the Northern, Eastern, and Western regions. The East and Northwest regions showed bimodal trends, and we were unable to associate local factors that could justify these changes. Although current scientific knowledge does not fully explain the determinants of these spatial disparities, similar regional variations have been documented in previous studies, such as those by Calderon et al., and in a prior investigation by our group in the state of Rio Grande do Sul [20,30].

The etiology of gastroschisis is widely recognized as multifactorial, with environmental variables playing a crucial role in the fluctuations of its incidence and prevalence. The hypothesis that external exposures and exogenous factors may influence the epidemiological behavior of this malformation has been widely discussed in specialized literature. Environmental factors, such as exposure to herbicides and electromagnetic fields, have already been described [8,31,32]. These factors, in theory, could explain the variations observed among the different health MRs analyzed in this study [8,32].

Another environmental factor to be considered is the COVID-19 pandemic that occurred from 2020 to 2023, but it did not have a significant influence on the annual incidence of gastronomic research as reported in a previous study in California [33].

The mortality rate was 36%. Moreover, nearly all deaths (98%) occurred within the first year of life. These findings corroborate previous investigations conducted in other states of southern Brazil [20]. This mortality rate reflects the typical scenario in developing countries. In Brazil, studies by authors such as Alves and Bilibio have reported variations, ranging from 14.9% to values exceeding 50% [17,20,34,35]. Globally, gastroschisis case fatality shows marked disparities. Rates as high as 98% have been recorded in Uganda [9], while in the United States, Allman and Georgeades, the reported case fatality is below 10% [4,8]. In this study, mortality rates remained stable at approximately 36%, a figure expected for the region, yet significantly distant from those in developed countries, such as the United States, where mortality rates have remained below 10% since the 1990s [18,36].

### 4.1. Maternal Characteristics: Maternal Age, Marital Status, Educational Level, and Prenatal Care Visits

In this study, the annual incidence of gastroschisis was significantly higher among younger mothers (*p* < 0.001; 95% CI: 1.95–2.15). Women under 25 years of age accounted for 77% of the cases (rate of 5.61 per 10,000 live births), a proportion markedly higher than the 37% observed in the general live birth population. These findings are consistent with data reported by Calderon [30] and a previous investigation conducted in Rio Grande do Sul [20]. Although the pathophysiological mechanisms linking young maternal age to gastroschisis have not yet been fully elucidated, this association remains an epidemiological constant across nearly all contemporary studies [6,28,37].

Regarding family structure, it was observed that mothers of children with gastroschisis were 16.5% more likely to lack a stable partner, with statistical significance. Approximately 60% of these women were without a partner at the time of birth, reflecting the predominance of young mothers in the study sample. Young mothers are often associated with less stable relationships and a higher incidence of unplanned pregnancies. Notably, the literature on the specific impact of paternal absence in cases of gastroschisis remains limited. A study by Kidane et al. [38] in Rwanda highlighted the additional challenges in managing this condition when social support is lacking. These findings underscore the need for a multidisciplinary approach that considers not only clinical factors but also the psychosocial context of these mothers [38].

Mothers of children with gastroschisis had significantly lower levels of education compared to the general population (*p* < 0.001; 95% CI: 1.14–1.22). A higher prevalence of women who had not completed 12 years of education was observed. In the Brazilian system, this corresponded to the completion of high school. This educational deficit is closely linked to the identified age profile, as early motherhood often disrupts the academic trajectory. Low maternal education, combined with young maternal age, constitutes a vulnerability scenario that may affect both early access to prenatal care and the understanding of the complex care required for the malformation. Low maternal education has also been reported in Brazil in a previous study by Regadas [39].

Regarding obstetric care, it was found that mothers of children with gastroschisis attended significantly fewer prenatal consultations compared to the control group (*p* < 0.001; 95% CI: 1.38–1.87). This finding revealed a significant gap in healthcare delivery. Given that gastroschisis is routinely diagnosed through prenatal ultrasonography, closer and more frequent follow-ups are warranted. However, the lower number of consultations observed may be largely explained by the high rate of prematurity identified in this sample, which reduces the total duration of gestational care. This deficit in prenatal care among gastroschisis cases corroborates findings reported by Regadas in other Brazilian contexts, highlighting a persistent challenge within the country’s public health system [39].

### 4.2. Pregnancy Characteristics: Type of Pregnancy, Gestational Age, and Mode of Delivery

This study had a predominance of singleton pregnancies, accounting for 97.7% in both the case group and the general live birth population. Consequently, no statistically significant difference was identified for this variable. The occurrence of gastroschisis is independent of pregnancy differences (singleton or multiple births). This finding is consistent with results reported by Oliveira and other studies, reinforcing the evidence that twinning is neither a risk nor a protective factor for the development of this malformation [39,40].

In contrast, prematurity emerged as most strongly associated with gastroschisis. While the incidence of preterm births (<37 weeks) in the general live birth population was 10.7%, this rate rose sharply to 57.7% among gastroschisis cases. This strong correlation corroborates findings from previous investigations [6,20,39] and may be attributed both to medically indicated preterm delivery—aimed at minimizing damage to the exposed intestinal loops due to prolonged contact with amniotic fluid—and to premature rupture of membranes and fetal distress, which are frequently associated with this condition. 

Analysis of the mode of delivery revealed that cesarean section was the predominant method among gastroschisis cases, occurring in 84.3% of births. This was significantly higher than in the general population (*p* < 0.001; 95% CI: 2.42–3.93). Of note, Paraná exhibits a high baseline cesarean rate (63.4%). However, the incidence of cesarean among gastroschisis cases is even more pronounced. This trend toward highly medicalized deliveries is consistent with the broader Brazilian context, as reported by several authors [39,41]. In the case of gastroschisis, the preference for surgical delivery may be associated with the need to coordinate immediate postnatal surgical repair, as well as with the high prevalence of prematurity and fetal distress—factors that often prompt the early termination of pregnancy via abdominal delivery.

### 4.3. Neonatal Characteristics: Race/Ethnicity, 1-Minute Apgar Score, 5-Minute Apgar Score, Birth Weight, and Sex

No statistically significant disparities were identified in terms of race/ethnicity between the groups analyzed. Similarly, the sex of the infants with gastroschisis showed no association with the occurrence of the malformation (*p* = 0.82; 95% CI: 0.90–1.08). These findings are consistent with previous investigations and suggest that the distribution of the condition in Paraná occurs independently of these variables, presenting a homogeneous epidemiological pattern with respect to neonatal demographic profiles [20,39,40].

Newborns with gastroschisis showed a significantly higher frequency of low birth weight (<2500 g) compared to the control population (*p* < 0.001; 95% CI: 6.82–7.80). This finding indicated a sevenfold increased risk of low birth weight in this group, corroborating evidence from the literature [6,34,40]. This disparity may be attributed to the strong association between the malformation and preterm birth. Additionally, it may suggest the occurrence of intrauterine growth restriction (IUGR), a phenomenon frequently linked to gastroschisis due to chronic loss of proteins and nutrients from the exposed bowel loops into the amniotic fluid.

Assessment of neonatal vitality revealed significantly impaired Apgar scores at both the first and fifth minutes among newborns with gastroschisis (*p* < 0.001). While the risk of low immediate vitality (1st minute) was four times higher (*RR* 4.09), the probability of maintaining scores below 8 at the fifth minute was nine times higher (*RR* 9.30; 95% CI: 7.78–11.12) compared to the control group. These results are consistent with the literature and indicate not only a challenging extrauterine transition but also persistent clinical instability in the first minutes of life. This neonatal vulnerability may reflect inflammatory visceral exposure and the high prevalence of prematurity, requiring immediate interventions and advanced resuscitation support in the delivery room [39,40,41].

### 4.4. Study Limitations

This study has some limitations. In particular, limitations inherent to the use of secondary databases are subject to variations in data quality and potential errors in the diagnostic coding of gastroschisis. However, these limitations were likely mitigated by the population-based nature of the sample, encompassing a substantial volume of records over a twelve-year period (2013–2024). The use of population-based data lends robustness to the findings and minimizes the impact of isolated inconsistencies, allowing for a reliable representation of the epidemiological reality of the malformation in the state of Paraná.

Another limitation is that maternal variables (young maternal age, low level of education, single marital status, and lower frequency of prenatal care) and neonatal variables (prematurity and low birth weight) are strongly interconnected, reflecting the epidemiological profile of gastroschisis. The coexistence of these factors makes it difficult to dissociate their individual effects on the outcome. Thus, the associations found may represent a complex of correlated factors, whose direct influence could only be fully isolated through advanced statistical modeling.

In this context, the absence of multivariate adjustment may leave the results susceptible to residual confounding. Furthermore, the use of polynomial regression introduced a potential risk of overfitting, where the model might have captured noise rather than the underlying trend. Consequently, while the observed patterns are clinically relevant, they warrant validation through larger datasets and more parsimonious statistical approaches in future studies.

## 5. Conclusions

This study demonstrated a significant 39.5% reduction in the prevalence of gastroschisis in the state of Paraná between 2013 and 2024. In contrast, the lethality of the malformation remained stable and high, at 36.5%, with nearly all deaths occurring within the first year of life. Although fewer infants with gastroschisis are being born in Paraná, the severity of cases or the clinical management required to prevent deaths remains a challenge.

The identified epidemiological profile reveals that gastroschisis in Paraná is strongly associated with maternal vulnerability, predominantly affecting young women under 25 years of age, without a partner, with low education levels and insufficient prenatal care. From a clinical-obstetric point of view, the condition occurs more frequently in singleton pregnancies, with high rates of prematurity and a notably high frequency of cesarean sections (84.3%). At the neonatal level, affected newborns have a higher risk of low birth weight and immediate impaired vitality, evidenced by low Apgar scores at one and five minutes.

These findings highlight the importance of continuous monitoring of epidemiological and mortality trends. Furthermore, improved public policies focused on reproductive planning, strengthening pediatric surgical support, and high-complexity neonatal care are likely necessary. Further studies are essential to elucidate the specific etiologies underlying the geographic variations in this condition in the state of Paraná.

## Figures and Tables

**Table 1:** Annual Incidence and Mortality Rates of Gastroschisis in Paraná, Brazil (2013–2024).

Annual Incidence in Paraná				Mortality in Paraná				
Year	Cases	Live Births	Rate ×	Deaths		Rate ×	Months	Months
			10,000			10,000	0–12	>12
2013	61	155,758	3.92	23	37.70%	1.48	23	0
2014	59	159,915	3.69	24	40.70%	1.5	23	1
2015	44	160,947	2.73	21	47.70%	1.3	20	1
2016	53	155,066	3.42	22	41.50%	1.42	22	0
2017	43	157,701	2.73	12	27.90%	0.76	12	0
2018	35	156,201	2.24	12	34.30%	0.77	12	0
2019	36	153,469	2.35	17	47.20%	1.11	15	2
2020	42	146,291	2.87	7	16.70%	0.48	7	0
2021	29	141,976	2.04	6	20.70%	0.42	6	0
2022	29	140,637	2.06	12	41.40%	0.85	12	0
2023	29	139,836	2.07	14	48.30%	1	14	0
2024	31	130,930	2.37	9	29.00%	0.69	9	0
2013–2024	491	1,798,727	2.73	179	36.50%	1	175 (97.7%)	4 (2.2%)

Source: SINASC/SUS, SIM 2013–2024.

**Table 2:** Annual Incidence and Mortality Rates and Trends of Gastroschisis in Paraná and Its Health Macroregions (MRs), 2013–2024.

Trends in Annual Incidence of Gastroschisis by Health Macroregion (MR) of Paraná				
Location	Regression Model	*R* ^2^	*p*-Value	Trend
Paraná	y = 0.0142x^2^ − 57.409x + 58120	0.97	<0.001	↓
Northern MR	y = −0.0053x^3^ + 31.859x^2^ − 64395x + 4 × 10^7^	0.74	0.029	↓
Northwestern MR	y = −0.0373x^2^ + 150.78x − 152178	0.75	0.116	↑↓
Eastern MR	y = 0.0074x^3^ − 44.869x^2^ + 90516x − 6 × 10^7^	0.95	0.020	↓↑
Western MR	y = −0.0144x^3^ + 87.34x^2^ − 176313x + 1 × 10^8^	0.85	0.002	↓
Case fatality				
Paraná	y = 0.0013x^3^ − 7.9755x^2^ + 16097x − 1 × 10^7^	0.83	0.452	↑↓↑

Note: ↑: increasing trend; ↓: decreasing trend; ↓↑ or ↑↓: fluctuating trend. Source: SINASC/SUS, SIM 2013–2024.

**Table 3:** Maternal Characteristics: Maternal Age, Marital Status, Years of Education, and Prenatal Care Visits in Paraná, Brazil, 2013–2024.

Characteristic	Cases		Live Births		Rate per 10,000 Live Births			*p*-Value
								95% CI
Maternal Age								
10 to 19 years	196	39.9%	242,160	13.5%	8.09	5.61		<0.001
20 to 24 years	182	37.1%	431,038	24.0%	4.22		2.73	(4.53–6.90)
>25 years old	113	23.0%	1,125,514	62.5%	1.00			
Ignored	0		15					
Maternal Marital Status								
Without a partner	289	59.1%	760,583	42.5%	3.80			<0.001
With a partner	200	40.9%	1,027,672	57.5%	1.95			(1.29–1.49)
Ignored	2		10,472					
Maternal Education								
<12 years of education	428	87.7%	1,327,122	74.1%	3.23			<0.001
>12 years of education	60	12.3%	463,535	25.9%	1.29			(1.14–1.22)
Ignored	3		8070					
Prenatal Visits								
<7 visits	124	25.3%	281,751	15.7%	4.40			<0.001
>7 visits	366	74.7%	1,513,253	84.3%	2.42			(1.38–1.87)
Ignored	1		3723					
Total	491	1,798,727						

Source: SINASC/SUS, 2013–2024.

**Table 4:** Pregnancy Characteristics: Type of Pregnancy, Gestational Age, and Mode of Delivery in Paraná, Brazil, 2013–2024.

	Cases		Live Births		Rate per 10,000 Live Births			*p*-Value
								95% CI
Type of Pregnancy								
Singleton	476	97.7%	1,755,235	97.7%		2.71		0.94
Twin	11	2.3%	41,467	2.3%		2.65		0.53–1.77
Ignored	4		2025					
Gestational Age								
<37 weeks	275	57.3%	191,329	10.7%	14.37			<0.001
>37 weeks	205	42.7%	1,591,637	89.3%	1.29			4.93–5.76
Ignored	11		15,761					
Mode of Delivery								
Cesarean	412	84.3%	1,139,411	63.4%	3.62			<0.001
Vaginal	77	15.7%	657,799	36.6%	1.17			1.27–1.38
Ignored	2		1517					
Total	491		1,798,727					

Source: SINASC/SUS, 2013–2024.

**Table 5:** Neonatal Characteristics: Race/Ethnicity, 1-Minute Apgar Score, 5-Minute Apgar Score, Birth Weight, and Sex in Paraná, Brazil, 2013–2024.

	Cases		Live Births		Rate Per 10,000 Live Births			*p*-Value
								95% CI
Race/ethnicity								
White	363	74.5%	1,315,351	73.9%	2.76			0.78
							
Black	10	2.1%	48,074	2.7%	2.08			0.70–2.48
							
Yellow	2	0.4%	6535	0.4%	3.06			0.88
								0.22–3.62
Brown	112	23.0%	405,317	22.8%	2.76			0.99
								0.80–1.23
Indigenous	0	0.0%	5793	0.3%	0.00			0.21
								undefined
Ignored	4		17,657					
Apgar Score (1 min)								
0 to 7	244	49.8%	217,341	12.1%	11.23			<0.001
8 to 10	246	50.2%	1,576,090	87.9%	1.56			3.75–4.49
Ignored	1		5296					
Apgar Score (5 min)								
0 to 7	97	19.8%	38,308	2.1%	25.32			<0.001
8 to 10	392	80.2%	1,755,435	97.9%	2.23			7.77–11.10
Ignored	2		4984					
Weight								
<2500 g	313	63.7%	157,052	8.7%	19.93			<0.001
>2500 g	178	36.3%	1,641,093	91.3%	1.08			6.82–7.80
Ignored	0		582					
Sex								
Male	247	50.7%	921,144	51.2%	2.68			0.82
Female	240	49.3%	876,922	48.8%	2.74			0.90–1.08
Ignored	4		661					
Total	491		1,798,727					

Source: SINASC/SUS, 2013–2024.

## Data Availability

The data presented in this study are available from the corresponding author upon reasonable request.
